# The Efficiency, Predictability and Safety Between Custom-Q Femotsecond Laser In Situ Keratomileusis and Second (Visumax 800) Generation Keratorefractive Lenticule Extraction Surgery

**DOI:** 10.3390/diagnostics15050634

**Published:** 2025-03-05

**Authors:** Chia-Yi Lee, Shun-Fa Yang, Ie-Bin Lian, Hung-Chi Chen, Jing-Yang Huang, Chao-Kai Chang

**Affiliations:** 1 Institute of Medicine, Chung Shan Medical University, Taichung 40201, Taiwan; 2 Nobel Eye Institute, Taipei 10041, Taiwan; 3 Department of Ophthalmology, Jen-Ai Hospital Dali Branch, Taichung 41265, Taiwan; 4 Department of Medical Research, Chung Shan Medical University Hospital, Taichung 40201, Taiwan; 5 Institute of Statistical and Information Science, National Changhua University of Education, Chunghua 50007, Taiwan; 6 Department of Ophthalmology, Chang Gung Memorial Hospital, Linkou 33305, Taiwan; 7 Center for Tissue Engineering, Chang Gung Memorial Hospital, Linkou 33305, Taiwan; 8 Department of Medicine, Chang Gung University College of Medicine, Taoyuan 33305, Taiwan; 9 Department of Optometry, Da-Yeh University, Chunghua 51591, Taiwan

**Keywords:** keratorefractive lenticule extraction, visumax 800, femtosecond laser in situ keratomileusis, uncorrected distance visual acuity, spherical equivalent

## Abstract

**Background/Objectives**: To evaluate the postoperative outcomes between the second-generation keratorefractive lenticule extraction (KLEx) surgery and femtosecond laser in situ keratomileusis (FS-LASIK). **Methods**: A retrospective cohort study was conducted and subjects received second-generation KLEx and FS-LASIK surgeries were enrolled. A total of 124 and 102 eyes were selected into the second-generation KLEx and FS-LASIK groups after exclusion. The primary outcomes were the postoperative uncorrected distance visual acuity (UDVA), spherical equivalent (SE), amount of astigmatism, and best-correct visual acuity (BCVA). The independent *t*-test was applied to compare the primary outcomes between groups. **Results**: The mean UDVA three months postoperatively showed insignificant differences between the two groups (*p* = 0.999). At the final visit, there were 113 (91.12%) and 96 (94.12%) subjects who reached UDVA 20/20 in the FS-LASIK and second-generation KLEx groups and the difference was statistically insignificant (*p* = 0.455), and the second-generation KLEx group illustrated a higher UDVA improvement (*p* = 0.046). The SE three months postoperatively showed insignificant difference between groups, whether the absolute value or the ratio of SE within ±0.50 D or ±1.00 D (all *p* > 0.05). The vector analysis indicated that the difference vector (DV) was significantly lower in the second-generation KLEx group (*p* = 0.033). The ratio of loss of more than 1 line BCVA showed insignificant differences between the two groups (all *p* > 0.05). In addition, the risk of postoperative dry eye disease (DED) was significantly higher in the FS-LASIK group (*p* = 0.031). **Conclusions**: The efficiency and predictability between second-generation KLEx and FS-LASIK surgeries are similar, while more DED occurred after FS-LASIK surgery.

## 1. Introduction

Refractive surgeries have been used to correct the refractive error on cornea surface in the past decades [1,2]. The laser in situ keratomileusis has been performed for more than 22 years now, with acceptable postoperative results. The same is true for the photorefractive keratectomy [2]. The uncorrected distance visual acuity (UDVA) of 20/20 after the refractive surgery has been displayed in 92% of human-accepted laser in situ keratomileusis and 72% of human-accepted photorefractive keratectomy [3,4]. On the other hand, several postoperative complications like ocular foreign body sensation, superficial keratitis, and dry eye disease (DED) have been found in the corneal refractive surgeries, which may diminish postoperative vision and subject satisfaction [5].

The keratorefractive lenticule extraction (KLEx) is one keratorefractive surgery that was first performed around 2010 and was previously known as the small incision lenticule extraction This surgery reduced refractive error via extracting a corneal lenticule [6,7,8,9,10]. Compared to the preceding corneal refractive surgeries, the KLEx has the beneficial result of a small incision, which causes less postoperative DED [11,12]. Regarding the postoperative outcomes in previous articles, the first generation KLEx is similar to both the laser in situ keratomileusis and photorefractive keratectomy [13,14,15,16]. In addition, the postoperative astigmatism of first generation KLEx surgery displayed not-inferior results compared to the femtosecond laser in situ keratomileusis (FS-LASIK), although the wavefront-guided function is absent in first generation KLEx surgery [17,18].

The second generation of KLEx surgery was launched in 2023 [19], and this surgery represents a faster laser emission speed and the presence of an eye-tracking system which was absent in first-generation KLEx surgery [20,21]. Still, there was no research that evaluated the postoperative outcomes between the FS-LASIK and second-generation KLEx surgeries. Because the new technique used in the second-generation KLEx surgery may lead to better management of astigmatism [22], the surgical outcomes between these two refractive surgeries may be different, and this should be evaluated.

The purpose of the current study is to compare the postoperative outcomes of the FS-LASIK surgery and second-generation KLEx surgery. In addition, the postoperative complications between the two surgeries were also evaluated.

## 2. Materials and Methods

### 2.1. Subject Selection

This retrospective cohort study was operated at the Nobel Eye Institute, which possesses more than 20 clinics throughout Taiwan. The criteria for selecting subjects for the current study were the following (1) age from 20 to 55 years, (2) myopia greater than −1.00 diopter (D) but lower than −9.00 D under cycloplegia refraction, (3) received FS-LASIK surgery or second-generation KLEx surgery at the Nobel Eye Institute, and (4) followed-up at any branch of the Nobel Eye Institute after the refractive surgeries not less than three months later. The subjects were arranged for FS-LASIK surgery or second-generation KLEx surgery according to their preference after thorough discussion with the ophthalmologists and no contraindications (i.e., the corneal thickness not being adequate for second-generation KLEx surgery). On the other hand, these exclusion factors were utilized to increase the homogeneity of the two populations: (1) an initial best-correct visual acuity (BCVA) lower than 20/40, (2) the presence of severe corneal or retinal diseases including but not limited to microbial keratitis, central corneal opacity, keratoconus, macular hole, macula-involved retinal detachment and central retinal arterial occlusion, (3) presence of uncontrolled glaucoma, end-stage glaucoma, or uveitis, (4) refraction alteration for more than ±0.50 D in the previous year, (5) pregnancy in the last three months, and (6) received monovision (planning residual myopia) management. In addition, only the right eye of each individual was enrolled in this study. Of note, the lowest preoperative BCVA in both groups were 0.9 (20/22). On the other hand, the retinal disease referred to in the study population was retinal degeneration (all the eyes received focal retinal photocoagulation before the refractive surgeries). Also, the blood pressure and blood sugar of patients with hypertension and diabetes were well-controlled for at least 6 months. After our selection process, a total of 124 and 102 eyes were put into the FS-LASIK group and the KLEx group, respectively.

### 2.2. Surgery Technique

All the keratorefractive surgeries in the current study were performed by two experienced refractive surgeons (C.-Y.L. and C.-K.C.). The FS-LASIK was completed by one femtosecond laser device (Visuamax 500, Carl Zeiss, Göschwitzer Str., Jena, Germany) and another excimer laser device with Custom-Q function (EX500, Alcon Laboratories, Fort Worth, TX, USA). After fixing the cornea with the suction instrument, the femtosecond laser device made a corneal flap with flap diameters from 7.5 to 8.5. The corneal flap was then lifted by a spatula and the excimer laser emitted onto the stromal surface after the iris registration process by the excimer laser device. After excimer laser emission, the stromal surface was washed with normal saline and the corneal flap was replaced to the original site. A soft contact lens was then placed on the corneal surface and removed 1 h after the FS-LASIK surgery. The second-generation KLEx surgery was performed by a second-generation femtosecond laser device (Visuamax 800, Carl Zeiss, Göschwitzer Str., Jena, Germany). The optic zone of second-generation KLEx surgery ranged from 5.5 to 6.9 mm, and the incision was created at a length of 3.0 mm at 105 degrees. The angle kappa was highlighted by the software Visuamax 800 in accordance with data from optical biometry (IOL Master 700, Carl Zeiss, Göschwitzer Str., Jena, Germany), and the coaxial sight corneal light reflex method and corneal topography were applied to refine the centration position. The whole cornea was fixated by a suction ring after the centration, then the femtosecond laser was emitted. Surgeons used a specialized spatula to divide the upper and lower interface of the lenticule, and then the corneal lenticule was removed by specialized forceps. Postoperatively, levofloxacin and prednisolone eye drops were administered for one week, then changed to sulfamethoxazole and fluorometholone eye drops for another three weeks. The artificial tear was administered for two months after the surgeries.

### 2.3. Ophthalmic Exam

All the subjects who received refractive surgeries experienced the same ophthalmic exams in all clinics of the Nobel Eye Institute. The preoperative exams included BCVA, cyclopegia refraction of both sphere power and cylinder power with the assistance autorefractor (KR-8900, Topcon, Itabashi-ku, Tokyo, Japan), central corneal thickness (CCT), corneal cylinder power, spherical aberration, total higher order aberrations, and pupil diameter by topographic instrument (TMS-5, Tomey Coporation, Nagoya, Aichi, Japan), and the axial length (AXL) and angle kappa was obtained via a biometry machine (IOL Master 700, Carl Zeiss, Göschwitzer Str., Jena, Germany). The Schirmer test with topical anesthesia was also performed before the surgery. The routine postoperative exams involved the UDVA and refraction measurement via the same devices as preoperative exams, and the BCVA was recorded three months after the refractive surgery. Regarding the surgical parameters, the optic zone, cap (2nd KLEx surgery) or flap (FS-LASIK) thickness, lenticule thickness (2nd KLEx surgery) or ablation depth (FS-LASIK), and the residual stromal thickness (RST) were collected. The medical records before the surgery, one day after the surgery, one week after the surgery, one month after the surgery, and three months after the surgery were obtained. For more details, the spherical equivalent (SE) was set as the sphere power plus half cylinder power in the current study, and the postoperative DED was set for the subjects with preoperative basic tear secretion of more than 10 mm, according to the Schirmer test, but who represented with mild superficial puntate keratitis and DED-related symptoms like dryness and ocular irritation after the refractive surgeries. We used the DED-related symptoms and the presence of mild superficial puntate keratitis as the occurrence of DED according to the criteria of grade I DED in the previous literature [23], and we regarded the grade II Oxford ocular surface stain as mild superficial puntate keratitis based on the previous literature [24,25]. The postoperative DED was evaluated at three months.

### 2.4. Statistical Analysis

The SPSS version 20.0 (SPSS Inc., Chicago, IL, USA) software was handled for statistical analysis of the study population. The Shapiro–Wilk test was handled to check the normality of all the eyes and normal distributions were found in every parameter (all the *p* > 0.05). The statistical power of the study population was 0.91, with an alpha value of 0.05 and a medium effect size, which was achieved by G∗power version 3.1.9.2 (Heinrich Heine Universität at Düsseldorf, Germany) software. The descriptive analysis was handled to represent the sex, age, topographic information, cycloplegia refraction, plus surgical information between the two groups. The independent *t*-test as well as Fisher’s exact test were performed to check the amount of the above factors between the two groups according to the each index’s property. Also, the independent *t*-test was used to investigate the amount of postoperative BCVA, UDVA, and SE between the FS-LASIK group and the 2nd KLEx group at different postoperative times. Concerning the trends of SE as well as UDVA alterations after refractive surgeries, the generalized estimate equation was used to verify the changes between the FS-LASIK and 2nd KLEx groups with the adjustments of sex, preoperative cycloplegia refraction, and age. Fisher’s exact test was used to calculate the numbers of eyes that reached a specific standard of postoperative BCVA, postoperative UDVA, and the postoperative SE between the FS-LASIK group and 2nd KLEx group. On the other hand, a vector analysis based on the Alpins method was also conducted [26], and the target-induced astigmatism (TIA), magnitude of error (ME), surgically induced astigmatism (SIA), correction index (CoI), angle of error (AE), and the difference vector (DV) between the FS-LASIK group and 2nd KLEx group were examined via the employment of independent *t*-tests. Notably, the SIA, TIA, and DV were displayed in arithmetic mean form. Finally, the dominant intraoperative and postoperative complications were attained from medical records, and Fisher’s exact test was then used to verify the proportion of intraoperative/postoperative complications between the two groups. Notably, the severe superficial puntate keratitis was defined as the Oxford ocular surface stain higher than IV [24]. A *p* value < 0.05 was interpreted as statistically significant, a *p* value over 0.999 was recounted as *p* > 0.999, and a *p* value under 0.001 was recounted as *p* < 0.001 in the current study.

## 3. Results

The preoperative parameters between the two groups are represented in Table 1. The mean age was 32.62 ± 9.44 and 31.25 ± 8.26 years old between the FS-LASIK and second-generation KLEx groups. The difference in age between the two groups did not reach a significant level (*p* = 0.252). Similarly, the sex distributions, systemic diseases, and retinal diseases between two groups showed insignificant difference (all *p* > 0.05). Regarding the ophthalmic indexes, the BCVA, cycloplegia refraction, topographic parameters including the spherical aberration, total higher order aberrations, and the Schirmer test results all demonstrated insignificant difference between the FS-LASIK and second-generation KLEx groups (all *p* > 0.05). The surgical data showed insignificant difference between the FS-LASIK and second KLEx groups (all *p* > 0.05) (Table 1).

Regarding the efficiency, the mean UDVA one day postoperatively was 0.09 ± 0.06 in the FS-LASIK group, which was significantly better than the 0.13 ± 0.07 in the second-generation KLEx group (*p* < 0.001). However, the mean UDVA three months postoperatively showed insignificant difference between the two groups (0.00 ± 0.03 versus 0.00 ± 0.04, *p* = 0.999). At the final visit, there were 113 (91.12%) and 96 (94.12%) subjects who reached 20/20 in the FS-LASIK and second-generation KLEx groups, and the difference was statistically insignificant (*p* = 0.455). In addition, the second-generation KLEx group illustrated a higher amount of visual improvement (*p* = 0.046) (Table 2).

Regarding the predictability, the SE performed one day postoperatively showed insignificant difference between the two groups (−0.27 ± 0.46 versus −0.22 ± 0.51, *p* = 0.440), and this similarity between the two groups persisted until the end of follow-up (−0.14 ± 0.07 versus −0.15 ± 0.10, *p* = 0.395) (Table 2). Also, the ratio of SE within ±0.50 D or ±1.00 D showed insignificant difference between the two groups (all *p* > 0.05) (Table 2), and the postoperative change in SE showed insignificant difference between the two groups (*p* = 0.538) (Table 2). The vector analysis indicates that the DV was significantly lower in the second-generation KLEx group than the FS-LASIK group (*p* = 0.033), while other parameters showed insignificant difference (all *p* > 0.05) (Table 3).

**Figure 1:**
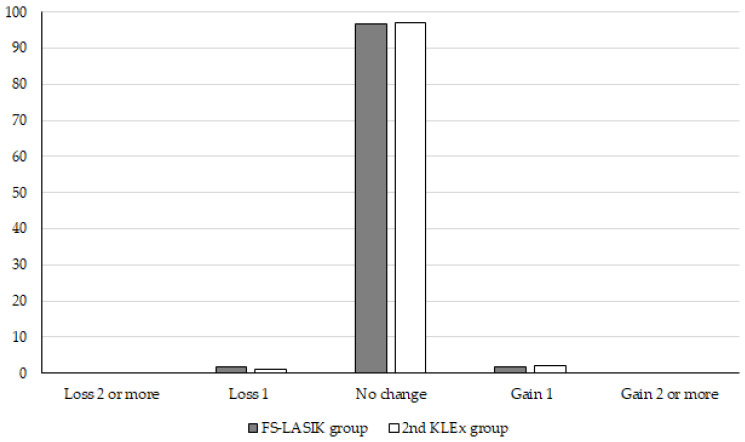
The postoperative best-corrected visual acuity status of the two groups. FS-LASIK: femtosecond laser in situ keratomileusis, KLEx: keratorefractive lenticule extraction.

Regarding the safety, the ratio of loss of more than 1 line BCVA showed insignificant difference between the two groups (*p* = 0.679) (Figure 1). Concerning the intraoperative/postoperative complications, the most common was postoperative DED, which occurred in 22 and 8 eyes in the FS-LASIK group and second-generation KLEx group, respectively, indicating that the risk of postoperative DED was significantly higher in the FS-LASIK group than the second-generation KLEx group (*p* = 0.031). The other intraoperative or postoperative parameters did not illustrate significant difference between the two groups (all *p* > 0.05) (Table 4).

## 4. Discussion

In the current study, the postoperative UDVA was better in the FS-LASIK group than in the second-generation KLEx group one day postoperatively, while the final UDVA showed insignificant difference. Moreover, the postoperative refraction showed insignificant difference between the FS-LASIK and the second-generation KLEx surgeries. On the other hand, the risk of postoperative DED was significantly higher in the FS-LASIK surgery than in the second-generation KLEx surgery.

The postoperative UDVA showed insignificant difference between the FS-LASIK group and the second-generation KLEx group at the final visit, and the degree of UDVA improvement was significantly better in the second-generation KLEx surgery. In the previous study, the postoperative UDVA was fair in the patients who received FS-LASIK surgery [27]. Also, the good postoperative UDVA was observed in the subjects who received second-generation KLEx surgery [28]. Nevertheless, the two studies were not conducted in the same time period with the same population, and thus the comparability between them may not be adequate. To our knowledge, the findings of the current study may be a preliminary effort at comparing the postoperative outcomes of FS-LASIK and the second-generation KLEx surgeries in the same settings and conditions. In addition, the preoperative refraction, topographic parameters, and visual acuity showed insignificant difference between the two groups, so the effect of different baseline conditions on our results may be minimal. Furthermore, we adjusted the age, sex and initial refractive status in the multivariable analysis to evaluate the trend of improvement. Consequently, the degree of improvement should indeed be better in the second-generation KLEx surgery than the FS-LASIK surgery. The worse UDVA one day postoperatively in the second-generation KLEx group may have been due to the high-frequency laser scanning of the second-generation KLEx surgery [19], which may have resulted in higher total laser energy and subsequent higher corneal edematous status. However, the percentage of subjects reaching the UDVA of 20/20 were even numerically higher in the second-generation KLEx group at the final visit. This discordance demonstrates that the effect of high laser energy on the cornea is transient. Also, a previous study showed that the optical density was higher in KLEx surgery than LASIK during the early postoperative period, which became lower than LASIK one month postoperatively [29]. The findings of the current study correspond to previous studies and may indicate that the efficiency of second-generation KLEx surgery is fair.

Regarding the postoperative refractive status between the FS-LASIK and second-generation KLEx surgery, the SE showed a not-significant difference between the two groups throughout the follow-up period. The previous study illustrated that the 3 month follow-up for the refractive surgery could be adequate [20], and the 3 month postoperative SE of the two groups presented a not-significant difference, implying that the final refractive outcomes between the FS-LASIK group and the second-generation KLEx surgery were close in the current study. Also, the trend of SE change showed an insignificant difference in the two groups, which indicates that the refractive recovery in the two types of refractive surgeries showed insignificant difference. The vector analysis presented grossly similar results between the two groups in which only the DV was significantly better in the second-generation KLEx surgery compared to the FS-LASIK surgery. There were few studies to support this phenomenon. The results may be a bit contrary to previous views of KLEx and FS-LASIK: the FS-LASIK has the iris registration function, which can compensate the cyclorotation at supine position and lead to better astigmatism management than KLEx [17]. Although the second-generation KLEx surgery can register the corneal apex and angle kappa [30], it cannot compensate the cyclotorsion. The possible explanations are that the eye-tracking system in second-generation KLEx surgery can manage the astigmatism as effectively as FS-LASIK in the real world, and the amount of TIA was higher in the FS-LASIK group, which caused a higher value of DV. Still, the differences in DV between the two groups were clinically insignificant. The mean astigmatism amount in the current study was around −1.50 D, and whether the predictability of FS-LASIK surgery and second-generation KLEx surgery are also similar in the high-astigmatism status requires further validation.

Regarding the safety aspect of the FS-LASIK surgery and the second-generation KLEx surgery, most of the subjects who received the FS-LASIK and second-generation KLEx surgery retained the preoperative BCVA three months after the surgery. Concerning the three patients could not remain in the preoperative BCVA, all of them having lost one line of BCVA and their current BCVA being 0.9. This may indicate that the BCVA loss of the two surgeries was moderate and may not cause significant visual impairment. The intraoperative complications demonstrated insignificant difference between the two groups, while the postoperative DED presented a higher risk in the FS-LASIK group than the second-generation KLEx group. The development of postoperative DED was not uncommon in the patients who received the LASIK procedure [31], with an incidence of about 20% at 3 months postoperatively [32]. Compared to the LASIK surgery, the KLEx surgery demonstrated a lower postoperative DED risk [33]. In the current study, the incidence of postoperative DED in FS-LASIK group was about 17.74% which was three-fold higher than the postoperative DED in the second-generation KLEx group and similar to the results in previous studies [32]. Actually, the rate of superficial puntate keratitis was three-fold in the FS-LASIK group compared to the second-generation KLEx group, despite the fact that we found no significant difference between groups. Overall, the results further highlight the risk of DED in LASIK surgery compared to the KLEx surgery.

Concerning the postoperative outcomes of the two keratorefractive surgeries in the current study compared to other refractive surgeries, the UDVA of the FS-LASIK surgery was 0.00, which is similar to the UDVA of LASIK or FS-LASIK in earlier publications [34,35]. In addition, the UDVA 3 month postoperative of the second-generation KLEx surgery also presents a similar value compared to the second-generation KLEx surgery in preceding article [28]. When it comes to the postoperative refraction, 100% of the subjects who received second-generation KLEx surgery revealed a postoperative SE within ±1.00 D, and 87.25% of subjects who received second-generation KLEx surgery revealed a postoperative SE within ±0.50 D, which is similar to the postoperative SE in subjects who received LASIK and the KLEx surgery [34,36]. On the other hand, 100% of subjects who received FS-LASIK surgery revealed a postoperative SE within ±1.00 D and 87.10% of subjects who received FS-LASIK revealed a postoperative SE within ±0.50 D, results which were also compatible to the LASIK surgery and the first-generation KLEx surgery according to the previous studies [7,10,13]. In addition, the safety indexes of the second-generation KLEx surgery and FS-LSIK surgery were 0.99, which is comparable to the 1.06 safety index of LASIK in the earlier research [37]. The results may imply that the surgical qualities of the second-generation KLEx surgery and FS-LASIK surgery in our institution are above average.

There are certain limitations to the current study. First, the retrospective design of the current study would reduce the integrity of analysis results and the homogeneity of the study population compared to the prospective one. Second, we did not measure the topographic change and AXL alteration after the refractive surgery, which are important postoperative factors for the corneal and refractive condition. In addition, we did not evaluate the DED with a systemic approach that should include the DED questionnaire, tear break-up time, Schirmer test, and ocular surface staining. Consequently, the postoperative DED in the current study may have had lower accuracy. Finally, the preoperative higher-order aberrations were not thoroughly measured, and the postoperative higher-order aberrations were not measured, thus the analysis of higher-order aberrations, which is an important issue when comparing refractive surgeries, cannot be performed.

## 5. Conclusions

In conclusion, the second-generation KLEx surgery and FS-LASIK surgery presented similar postoperative UDVA and visual recovery. Furthermore, the astigmatism correction between the two surgeries was also similar, while more postoperative DED would occur after FS-LASIK surgery. Consequently, patients with previous DED may choose the second-generation KLEx surgery to prevent DED progression. Further large-scale prospective studies to evaluate astigmatism correction in a high-astigmatism population and the postoperative outcomes in a large angle kappa population between these two refractive surgeries is mandatory.

## Figures and Tables

**Table 1:** Basic characters of the two groups.

Characters	FS-LASIK Group (*n* = 124)	2nd KLEx Group (*n* = 102)	*p*
Age	32.62 ± 9.44	31.25 ± 8.26	0.252
Sex (male:female)	51:73	46:56	0.590
Systemic disease			0.973
Hypertension	2	1	
Diabetes mellitus	1	1	
Others	2	2	
Retinal disease	2	2	0.844
BCVA (LogMAR)	0.00 ± 0.02	0.00 ± 0.03	0.999
Cycloplegic refraction (D)			
Sphere	−5.24 ± 2.01	−4.95 ± 2.26	0.309
Cylinder	−1.63 ± 0.68	−1.48 ± 0.56	0.076
SE	−6.06 ± 1.85	−5.69 ± 1.61	0.114
Topographic cylinder (D)	−2.07 ± 1.09	−1.87 ± 0.83	0.129
CCT (μm)	546.93 ± 25.69	550.21 ± 26.84	0.350
Angle kappa	0.17 ± 0.12	0.15 ± 0.15	0.267
Pupil diameter (mm)	4.02 ± 0.88	4.13 ± 0.76	0.321
Spherical aberration	0.14 ± 0.09	0.13 ± 0.09	0.408
Total higher order aberrations	0.30 ± 0.14	0.32 ± 0.16	0.318
Schirmer test (mm)	12.19 ± 3.74	11.82 ± 3.49	0.447
Optic zone (mm)	6.83 ± 0.52	6.91 ± 0.55	0.263
Cap/flap thickness (μm)	118.76 ± 7.97	116.83 ± 8.10	0.074
Lenticule thickness/ablation depth (μm)	121.95 ± 14.84	124.91 ± 13.69	0.125
RST (μm)	306.22 ± 20.56	308.47 ± 18.48	0.392

BCVA: best-corrected visual acuity, CCT: central corneal thickness, KLEx: keratorefractive lenticule extraction, *n*: number, SE: spherical equivalent, RST: residual stromal thickness, FS-LASIK: femtosecond laser in situ keratomileusis.

**Table 2:** Postoperative visual acuity and spherical equivalent between the two groups.

Outcome	FS-LASIK Group (*n* = 124)	2nd KLEx Group (*n* = 102)	P1	P2
UDVA (LogMAR)				
1 day	0.09 ± 0.06	0.13 ± 0.07	<0.001 *	
1 week	0.06 ± 0.05	0.05 ± 0.04	0.096	
1 month	0.02 ± 0.05	0.01 ± 0.03	0.064	
3 months	0.00 ± 0.03	0.00 ± 0.04	0.999	
≥20/25	100.00	100.00	0.999	
≥20/20	91.12	94.12	0.455	
Total change	0.09 ± 0.04	0.13 ± 0.04		0.046 *
SE				
1 day	−0.27 ± 0.46	−0.22 ± 0.51	0.440	
1 week	−0.21 ± 0.13	−0.18 ± 0.15	0.109	
1 month	−0.14 ± 0.13	−0.15 ± 0.14	0.579	
3 months	−0.14 ± 0.07	−0.15 ± 0.10	0.395	
≤±0.50 D	87.10	87.25	0.972	
≤±1.00 D	100.00	100.00	0.999	
Total change	0.13 ± 0.19	0.07 ± 0.24		0.538

KLEx: keratorefractive lenticule extraction, *n*: number, SE: spherical equivalent, UDVA: uncorrected distance visual acuity, FS-LASIK: femtosecond laser in situ keratomileusis. P1: the difference in postoperative parameters between the two groups. P2: the trend of postoperative parameter changes between the two groups after adjusting for age, sex, and preoperative cycloplegia refraction. * Denotes significant difference between the two groups.

**Table 3:** The vector analysis of astigmatism between the two groups.

Parameter	FS-LASIK Group (*n* = 124)	2nd KLEx Group (*n* = 102)	*p*
TIA	−1.52 ± 0.64	−1.39 ± 0.52	0.093
SIA	−1.33 ± 0.53	−1.24 ± 0.46	0.179
DV	0.43 ± 0.30	0.35 ± 0.25	0.033 *
ME	−0.19 ± 0.27	−0.16 ± 0.22	0.358
AE	2.38 ± 11.51	3.59 ± 16.46	0.532
CoI	0.88 ± 0.12	0.89 ± 0.11	0.518

AE: angle of error, CoI: correction index, DV: difference vector, KLEx: keratorefractive lenticule extraction, ME: magnitude of error, *n*: number, SIA: surgically induced astigmatism, TIA: target-induced astigmatism, FS-LASIK: femtosecond laser in situ keratomileusis. * Denotes significant difference between groups.

**Table 4:** Intraoperative and postoperative complication between the two groups.

Complication	FS-LASIK Group (*n* = 124)	2nd KLEx Group (*n* = 102)	*p*
Cap/flap tear	1	3	0.330
Epithelial defect	2	2	0.844
Severe superficial puntate keratitis	12	4	0.059
Dry eye disease	22	7	0.031 *
Corneal edema	0	0	0.999
Interface foreign body	1	0	0.549
Epithelial ingrowth	1	1	0.999
Diffuse lamellar keratitis	1	1	0.999
Microbial keratitis	0	0	0.999

*n*: number, KLEx: keratorefractive lenticule extraction, FS-LASIK: femtosecond laser in situ keratomileusis. * Denotes significant difference between the two groups.

## Data Availability

The data used in the current study is available from the corresponding author upon reasonable request.
